# Prediction of Potential Suitable Habitats for *Elaphodus cephalophus* in China Under Climate Change Scenarios

**DOI:** 10.1002/ece3.72194

**Published:** 2025-09-25

**Authors:** Huilin Liu, Qing Liu, Xiaojuan Cui, Jianjun Peng, Sini Zhou, Fuli Wang, Lizhen Zhong, Xia Wang, Haifeng Zheng, Chengzhong Yang, Ling Shen, Xudong Yuan, Lixia Chen, Chenglun Zhang

**Affiliations:** ^1^ School of Life and Health Sciences Hunan University of Science and Technology Xiangtan China; ^2^ Chongqing Jinfo Mountain National Nature Reserve Management Affairs Center Chongqing China; ^3^ Animal Biology Key Laboratory of Chongqing Education Commission of China, College of Life Sciences Chongqing Normal University Chongqing China

**Keywords:** climate change, habitat suitability, MaxEnt model, shared socioeconomic pathways (SSPs), species distribution modeling (SDM), ungulate

## Abstract

Global climate change exerts profound impacts on biodiversity and species distributions, potentially leading to habitat contraction and species extinction. As an endemic near‐threatened species designated under China's National Class II Protected Wildlife, the tufted deer ( *Elaphodus cephalophus* ) lacks comprehensive predictions regarding its future distribution under climate change. This study employs an optimized MaxEnt model integrated with 19 climatic variables and environmental factors (topography, vegetation, and anthropogenic disturbances) to systematically predict the species' potential habitat distribution across China. Through parameter screening of 248 combinations using the Kuenm package, the optimal model configuration achieved exceptional predictive accuracy (AUC = 0.977 ± 0.002). Key findings include: (1) Current suitable habitats span 145.98 × 10^4^ km^2^, predominantly clustered in the Sichuan‐Guizhou‐Yunnan mountainous regions and the Qinling‐Daba‐Wuling ranges; (2) Annual precipitation, elevation, slope, temperature annual range, NDVI, and temperature seasonality emerged as the variables that performed best in predicting habitat suitability for tufted deer; (3) Projected habitat areas under future climate scenarios will contract by 21.8%–28.4%, with shrinkage concentrated in eastern low‐elevation zones and expansion toward the eastern Qinghai‐Tibet Plateau; (4) Habitat centroids exhibit significant westward shifts, reaching 141.8 km under SSP585 (2090s). This study provided theoretical foundations for conserving *E. cephalophus* genetic resources and climate‐adaptive management, emphasizing the urgency to prioritize ecological corridor construction in western Sichuan‐southeastern Tibet.

## Graphical abstract

The study investigates the potential habitat distribution of the tufted deer ( *Elaphodus cephalophus* ) in China under current and future climate change scenarios. By employing an optimized MaxEnt model and integrating 19 climatic variables and environmental factors, the research identifies key limiting factors and projects significant habitat contraction and westward centroid shifts under future climate change. The findings emphasize the urgent need for conservation efforts to prioritize ecological corridor construction in western Sichuan–southeastern Tibet and highlight the importance of climate‐adaptive management strategies for this endangered species.

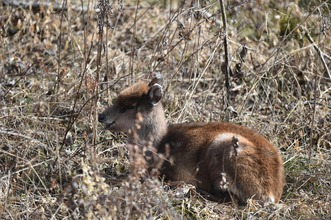

## 1 Introduction

Global climate change, recognized as one of the most critical ecological challenges of the 21st century, is profoundly altering species' distribution boundaries, phenological rhythms, and population dynamics through rising temperatures, shifting precipitation patterns, and increased frequency of extreme climatic events (Walther et al. 2002; Root et al. 2003). Studies indicate that biological communities are responding to climate warming by migrating toward higher latitudes and elevations, a phenomenon that has triggered systemic disruptions in ecosystem trophic cascades (Peng et al. 2002; Lenoir et al. 2008; Glennon et al. 2019). In this context, accurately assessing species' ecological niche responses has emerged as a central focus in conservation biology research.

To quantify the dynamic impacts of climate change on species distributions, ecologists have developed Species Distribution Models (SDMs), which analyze known species occurrence data alongside environmental variables to evaluate ecological niches and predict potential spatiotemporal distributions under current conditions and future climate scenarios (Yao et al. 2025). Common SDMs include the Maximum Entropy Model (MaxEnt), BIOCLIM, Ecological Niche Factor Analysis (ENFA), Genetic Algorithm for Rule‐set Prediction (GARP), Generalized Linear Models (GLM), and Generalized Additive Models (GAM) (Zhuang et al. 2024). Among these, the MaxEnt model, developed by Phillips et al. (2006), based on the principle of maximum entropy, has gained widespread use in ecological studies due to its ability to handle small sample sizes, low data requirements, and robust predictive accuracy under complex environmental conditions (Shi et al. 2023). MaxEnt has demonstrated unique advantages in invasive species control (Poudel et al. 2024; Waheed et al. 2024), endangered species conservation (Khattak et al. 2024; MacPherson et al. 2024), and disease transmission modeling (Shirzad et al. 2023; Neves et al. 2024).

**FIGURE 1:**
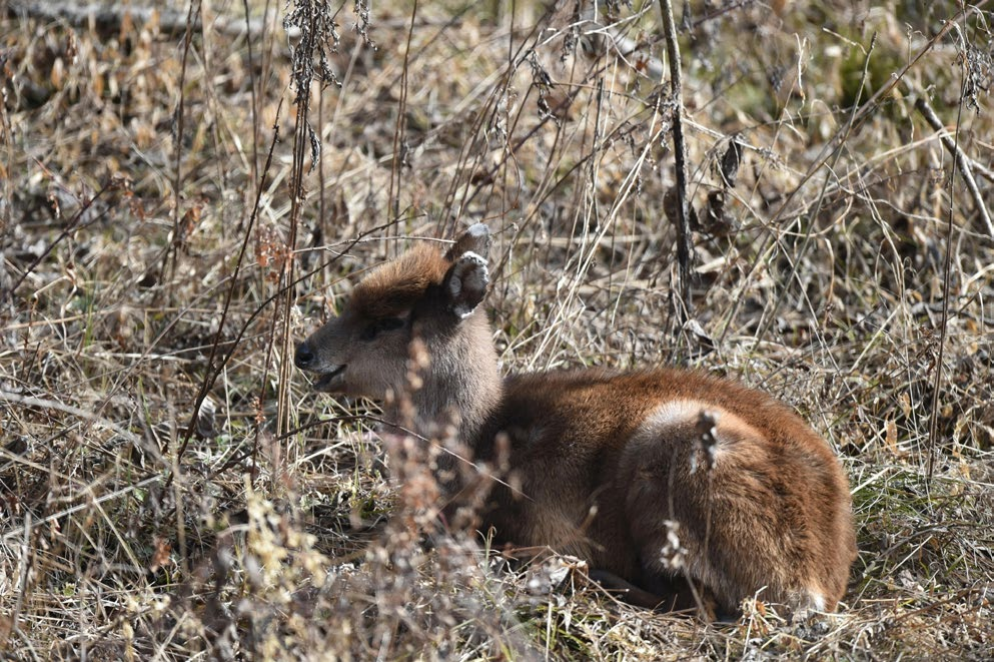
Juvenile Tufted Deer ( *Elaphodus cephalophus* ) in wild habitat. Image courtesy of Philip, Sichuan Zhuye International Travel Service, taken in the Tangjiahe National Nature Reserve, Sichuan Province, China.

The tufted deer ( *Elaphodus cephalophus* ) (Figure 1), also known as the black deer, is a small subtropical herbivorous mammal belonging to the Cervidae family. Primarily distributed across southern China's mountainous and hilly regions (Sheng and Lu 1982), its population has declined due to illegal hunting, habitat fragmentation, and anthropogenic disturbances. The species is classified as Near Threatened (NT) on the IUCN Red List and was designated as a National Class II Protected Wildlife in China in 2021.

However, our understanding of its precise nationwide distribution is fragmented, largely pieced together from localized surveys. While early research established foundational knowledge of its range through transect sampling (Sheng and Wu 1981; Sheng and Lu 1982), and subsequent studies employed advanced techniques like camera trapping and quadrat‐based analysis to investigate microhabitat selection and behavior in specific reserves (Zhang and Wei 2007; Liu et al. 2021; Lu et al. 2023), these efforts have inherently been geographically constrained. More recently, SDMs have been applied to the conservation of the species, yet these studies also suffer from a narrow spatial focus. For instance, a study modeled the species' climate‐driven distribution shifts but was limited to Hunan Province (Yang, Yang, et al. 2024). A key deficiency of such a geographically restricted approach is its inability to account for broader, cross‐provincial ecological corridors and potential climate refugia, which are essential for developing a robust national conservation strategy. Consequently, a systematic, nationwide assessment of the tufted deer's ecological niche and potential distribution under climate change is conspicuously absent, leaving a critical gap in large‐scale conservation planning.

To address this critical research gap, this study employs the Kuenm‐optimized MaxEnt model, integrating climatic and topographic variables, to predict the potential habitat suitability for *E. cephalophus* across China under current conditions and future climate scenarios. By identifying key environmental drivers and shifts in suitable habitats, the research addresses two scientific questions: (1) What are the spatial distribution patterns of *E. cephalophus* under current conditions and future climate scenarios? (2) What are the contraction, expansion, and stable zones of its distribution, as well as centroid shifts, under future climatic scenarios? The findings aim to provide theoretical support for the conservation and adaptive management of *E. cephalophus* genetic resources in China, particularly in the context of accelerating climate change.

## 2 Methods

### 2.1 Technical Workflow

**FIGURE 2:**
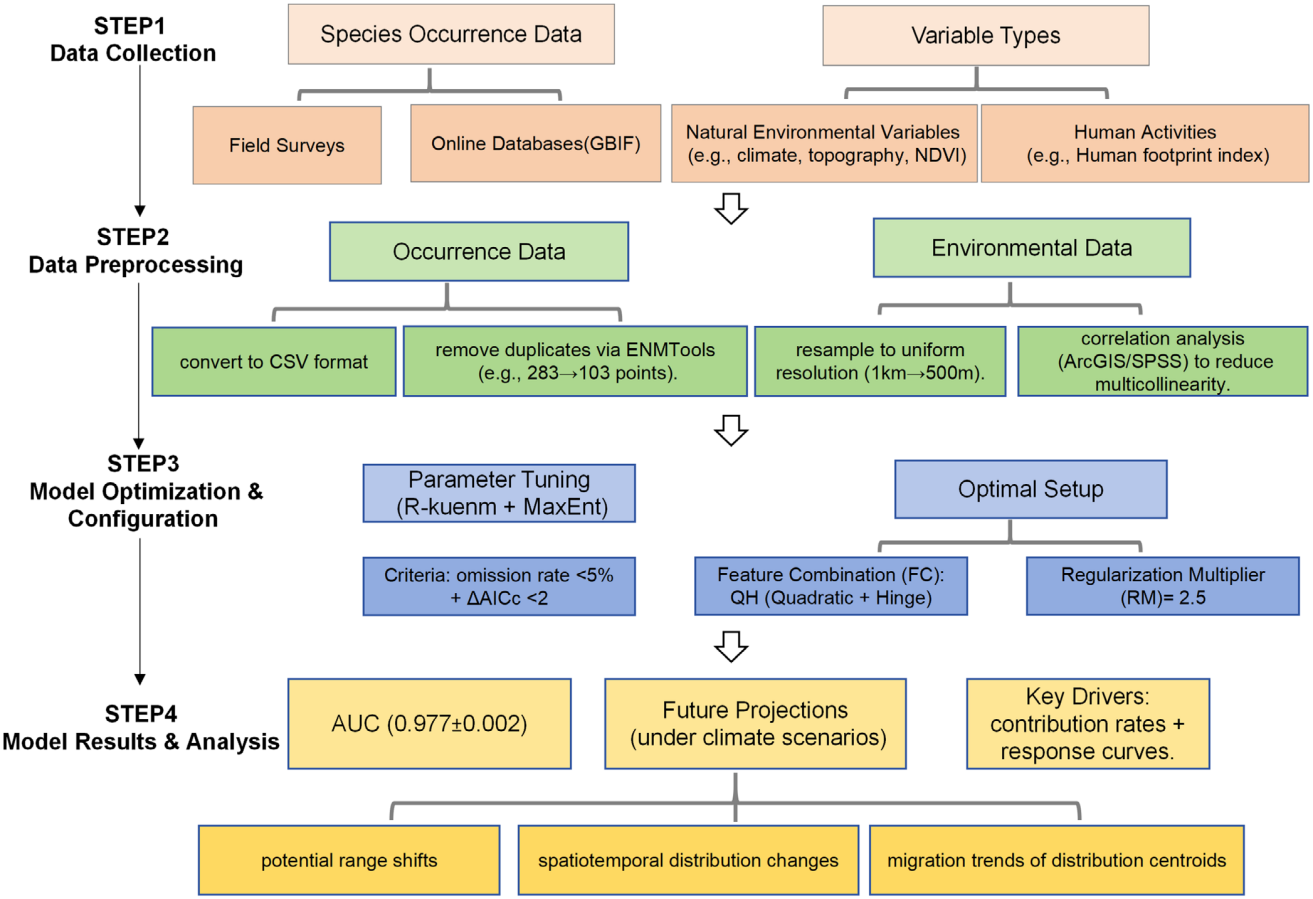
The technical approach and framework of this study are outlined as follows.

Based on the principles of the MaxEnt model and research objectives, we established a technical workflow to predict the current conditions and future suitable habitat distribution of *E. cephalophus* (Figure 2). The workflow consists of four sequential phases: (1) Data collection; (2) Data processing; (3) Model optimization and configuration; (4) Model results and analysis.

### 2.2 Species Occurrence Data Acquisition and Processing

The species distribution data for this study were derived from two sources: (1) Online databases: records retrieved from the Global Biodiversity Information Facility (GBIF; https://www.gbif.org, accessed on 11 December 2024); (2) Field surveys: To survey the geographic distribution of *E. cephalophus* , we employed a stratified sampling design for transect placement between 2023 and 2024. The study areas, encompassing natural reserves and typical forested areas in Chongqing and Hunan provinces, were stratified by altitudinal gradients and habitat types to ensure representative coverage. Within each stratum, transects were systematically established. Each transect had a fixed width of 50 m, and a minimum distance of ≥ 1000 m was maintained between adjacent transects to ensure spatial independence. Along the transects, the research team systematically searched for biological traces of *E. cephalophus* , such as feces and hair remnants, and recorded coordinates of activity hotspots.

**FIGURE 3:**
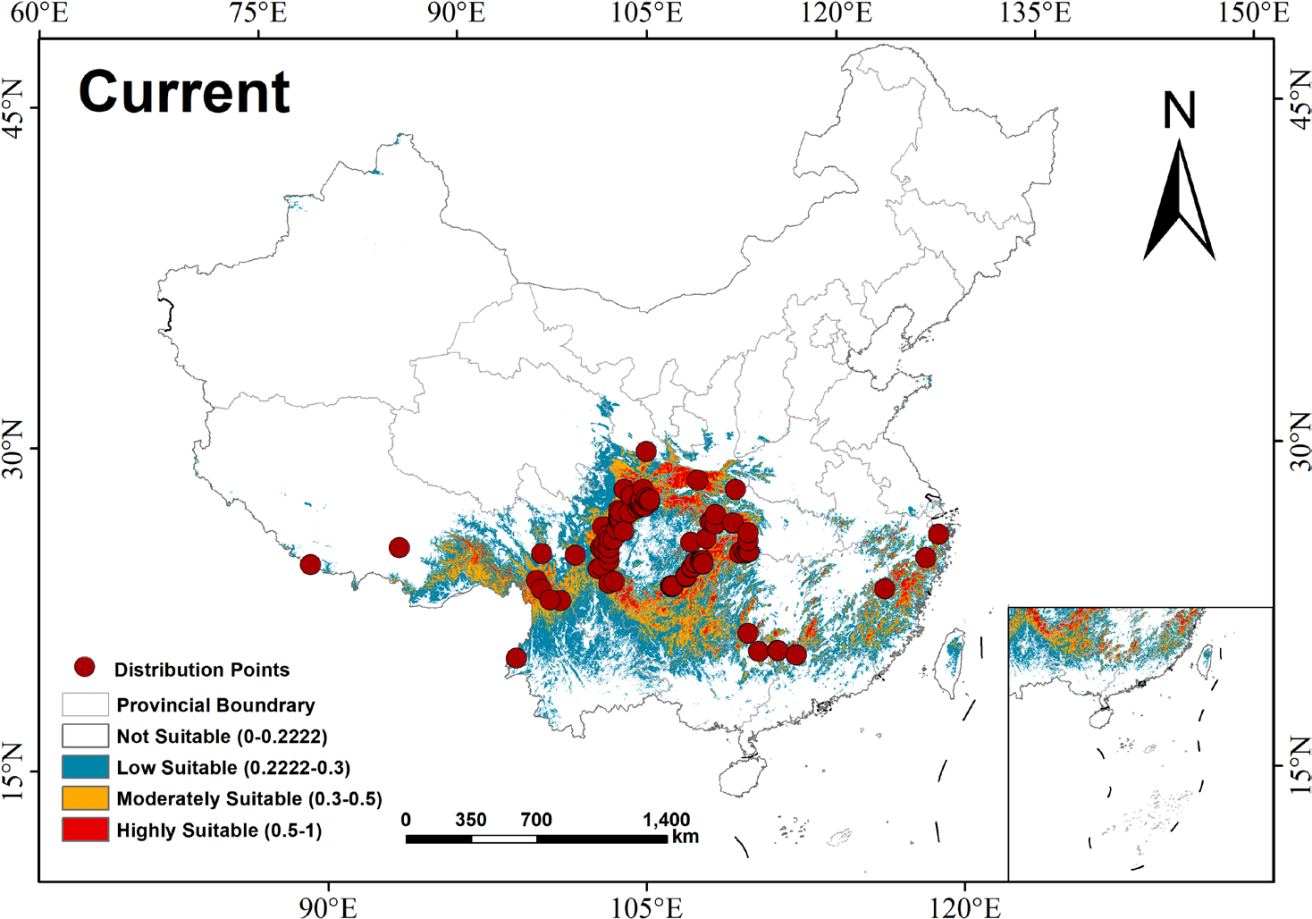
Current distribution points and suitable habitat distribution of *E. cephalophus* in China under current climatic conditions.

A total of 283 raw occurrence records were compiled. To minimize spatial autocorrelation among occurrence points and reduce model overfitting risks, duplicate records were eliminated using ENMTools.pl, ensuring only one occurrence point was retained per 1 km × 1 km grid cell. After spatial filtering, 103 spatially independent occurrence points were selected for modeling (Figure 3). All data were subsequently converted into CSV format to comply with MaxEnt model requirements.

### 2.3 Environmental Variable Data Acquisition and Processing

This study incorporated 24 environmental variables for modeling, including 19 bioclimatic variables, 3 topographic factors, NDVI, and a Human Footprint Index (Table 1). Current climate data (1970–2000) and future climate projections for the periods 2021–2040, 2041–2060, 2061–2080, and 2081–2100 were obtained from WorldClim (Version 2.1, https://worldclim.org/, accessed 13 December 2024). Elevation data (DEM) were sourced from the General Bathymetric Chart of the Oceans (GEBCO, https://www.gebco.net/, accessed 13 December 2024). Slope and aspect layers were derived from DEM using surface analysis tools in ArcGIS 10.8.2. The 2016 Global Human Footprint Index, integrating multidimensional socioeconomic factors (population density, land cover, infrastructure, and transportation networks) to quantify cumulative anthropogenic impacts (Di Marco et al. 2018), was acquired from Columbia University's Socioeconomic Data and Applications Center (SEDAC, https://sedac.ciesin.columbia.edu/, accessed 14 December 2024). NDVI data were provided by the National Earth System Science Data Center (https://www.geodata.cn, accessed 14 December 2024).

**TABLE 1:** Variables used in modeling and their contribution to the model.

Environmental variables	Variable description	Percent contribution (%)	Permutation importance (%)	Range of Suitability (SI > 0.5)
Bio1	Annual mean temperature (°C)	—	—	—
Bio2*	Mean diurnal range (mean of monthly (max temp–min temp)) (°C)	4.3	10.4	3.64~8.81
Bio3	Isothermality (Bio2/Bio7) (×100)	—	—	—
Bio4*	Temperature seasonality (standard deviation × 100)	7.5	55.9	591.83~772.18
Bio5	Max temperature of warmest month (°C)	—	—	—
Bio6	Min temperature of coldest month (°C)	—	—	—
Bio7*	Temperature annual range (Bio5‐Bio6) (°C)	9.2	5.3	25.93~29.76
Bio8	Mean temperature of wettest quarter (°C)	—	—	—
Bio9	Mean temperature of driest quarter (°C)	—	—	—
Bio10*	Mean temperature of warmest quarter (°C)	1.3	12.8	10.80~22.91
Bio11	Mean temperature of coldest quarter (°C)	—	—	—
Bio12*	Annual precipitation (mm)	33.2	0.7	727.79~1515.49
Bio13	Precipitation of wettest month (mm)	—	—	—
Bio14	Precipitation of driest month (mm)	—	—	—
Bio15*	Precipitation seasonality (coefficient of variation)	0.4	1.4	51.50~77.40
Bio16	Precipitation of wettest quarter (mm)	—	—	—
Bio17	Precipitation of driest quarter (mm)	—	—	—
Bio18	Precipitation of warmest quarter (mm)	—	—	—
Bio19	Precipitation of coldest quarter (mm)	—	—	
Asp*	Aspect (extract from DEM) (rad)	0.3	0.1	7.08~200.22
Ele*	Elevation (m)	18.7	9.7	1198.56~3612.90
Slo*	Slope (extract from DEM) (°)	14.5	0.1	> 25.19
Human*	Human disturbance index	2.7	1.9	0.13~0.37
NDVI*	Normalized difference vegetation index	7.9	1.8	0.68~0.89

*Note:* (*) Denotes that the variable is used for final modeling.

Future climate projections employed the Beijing Climate Center Climate System Model (BCC‐CSM2‐MR) from CMIP6, renowned for its robust performance in simulating temperature, precipitation, and atmospheric circulation patterns in China (Wu et al. 2019; Wang, Liu, et al. 2024). Four Shared Socioeconomic Pathways (SSPs) were selected: SSP126 (low emissions), SSP245 (medium emissions), SSP370 (high emissions), and SSP585 (extreme emissions). All environmental layers were resampled to 500 m resolution (500 m × 500 m) using ArcGIS 10.8.2 and clipped to China's administrative boundaries obtained from the National Geographic Information Public Service Platform (Tianditu, https://cloudcenter.tianditu.gov.cn, accessed 15 December 2024).

**FIGURE 4:**
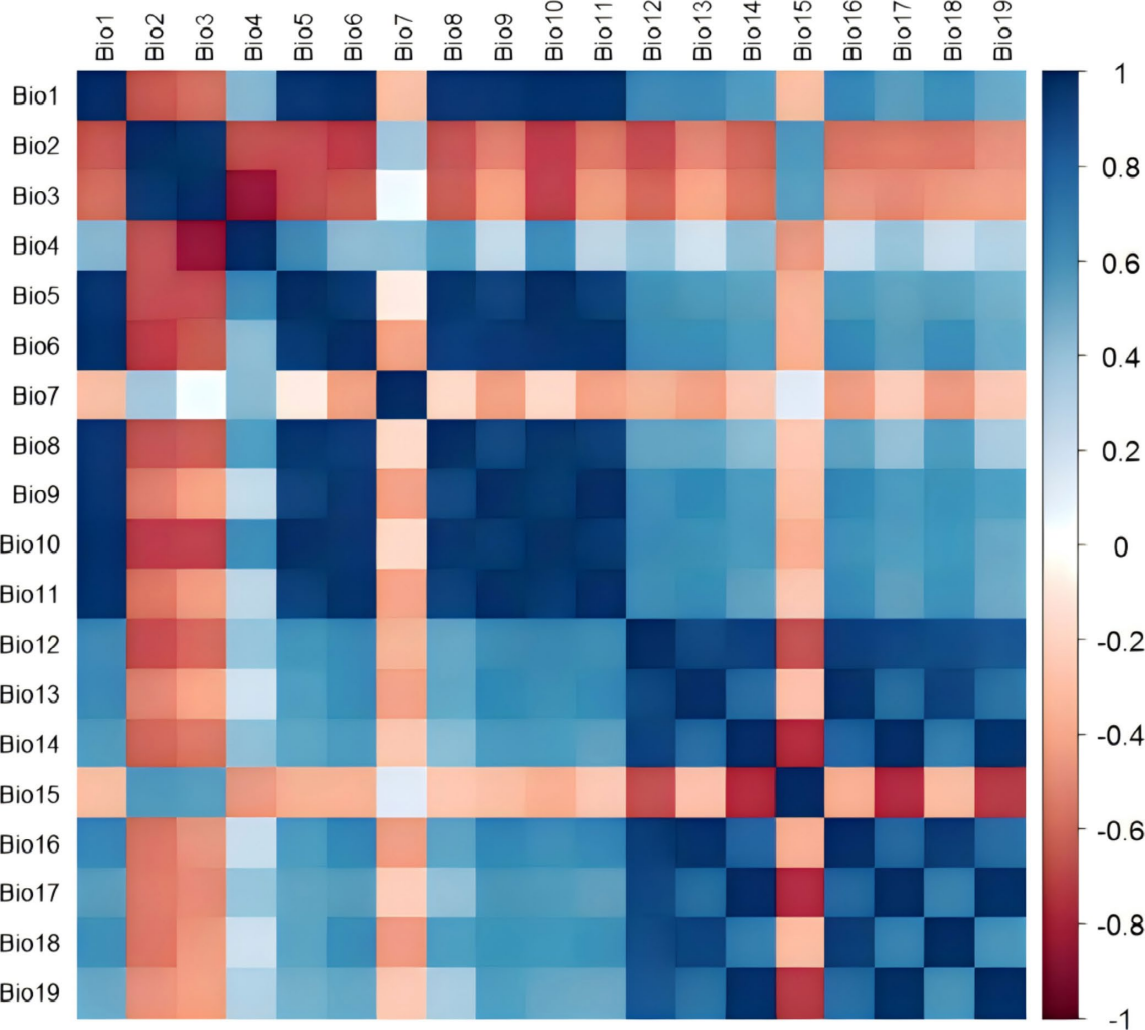
Correlation analysis of environmental variables.

To address multicollinearity and minimize model overfitting, environmental variable selection followed a three‐step protocol: (1) Jackknife tests in MaxEnt evaluated the contribution rates of 19 bioclimatic variables using 103 occurrence points; (2) Environmental attribute values at occurrence sites were extracted via ArcGIS 10.8.2, with Pearson correlation coefficients (|*r*|) calculated in SPSS (Table S1; Figure 4); (3) Variables with |*r*| < 0.8 were retained, while those with |*r*| ≥ 0.8 were filtered by prioritizing higher jackknife contribution rates (Burgos et al. 2020; Zhang et al. 2020). This process systematically reduced the initial set of 19 bioclimatic variables to 6 key climatic variables with low collinearity and high predictive power. These 6 selected climatic variables, along with the 5 non‐climatic variables (Elevation, Slope, Aspect, NDVI, and Human Footprint), constituted the final set of 11 predictors used for model construction. The five non‐climatic variables were assumed to remain stable across future scenarios (Table 1).

### 2.4 Species Distribution Model Optimization, Construction, and Evaluation

While ensemble models, which combine outputs from multiple algorithms, are often used to account for inter‐model variability, this study opted for a single‐model approach centered on a rigorously optimized MaxEnt model. This decision is grounded in MaxEnt's consistent high performance with presence‐only data and, more importantly, on the principle that a single, meticulously calibrated model can yield more reliable and ecologically interpretable results than an ensemble of unoptimized models (Syfert et al. 2013; Radosavljevic and Anderson 2014). Our approach prioritizes in‐depth parameter tuning to build the most robust and transparent model possible, ensuring that the resulting habitat suitability predictions are directly linked to specific, optimized parameters and are clearly interpretable for conservation planning. To address the inherent complexity of MaxEnt models that may compromise prediction accuracy, systematic parameter optimization was implemented to mitigate overfitting and enhance predictive performance (Morales et al. 2017; Wang, Liu, et al. 2024). The optimization workflow utilized R software v4.4.1 (https://www.r‐project.org/) with the Kuenm package (https://github.com/marlonecobos/Kuenm), which enables rigorous hyperparameter tuning. Two critical parameters—feature combinations (FC) and regularization multipliers (RM)—were combinatorially tested. The FC parameter included five feature types: Linear (L), Quadratic (Q), Product (P), Threshold (T), and Hinge (H), generating 31 potential combinations under the default LQPH configuration. The RM parameter was evaluated across eight values (0.5–4.0, increment = 0.5). This generated 248 unique parameter combinations (31 FC × 8 RM) for comprehensive model calibration.

Optimal model selection obeyed dual criteria: spatial omission rates ≤ 5% and minimal difference in corrected Akaike Information Criterion (ΔAICc < 2.0), following ecological niche modeling best practices (Cobos et al. 2019; Yang et al. 2025). The finalized model configuration employed FC = QH and RM = 2.5, achieving an AUC of 0.977 (±0.002 SD) through 10‐fold cross‐validation.

Model construction incorporated 103 spatially filtered occurrence points and 11 bioclimatic‐topographic variables. Key configurations included: (1) optimized FC and RM parameters; (2) generation of response curves and jackknife assessments for variable importance ranking; (3) random partitioning of 25% occurrence data for testing with 10 bootstrap replicates; and (4) activation of random seed initialization to ensure (Phillips et al. 2006). Model discrimination capacity was quantified using the AUC metric, where values ≥ 0.9 indicate excellent predictive performance (Swets 1988).

Habitat suitability classification adopted the Maximum Training Sensitivity‐Specificity (MTSS) threshold method, which minimizes commission and omission errors by balancing true positive rates (sensitivity) and true negative rates (specificity) (Liu et al. 2016). This approach demonstrates robustness against sampling bias and prevalence imbalances, outperforming arbitrary threshold selection methods (Kong et al. 2019; Huang et al. 2022). Suitability gradients were categorized as: not suitable (0–0.2222), low suitable (0.2222–0.3), moderately suitable (0.3–0.5), and highly suitable (0.5–1.0).

### 2.5 Habitat Suitability Dynamics and Centroid Shifts Under Future Climate Scenarios

Habitat dynamics and the range centroid shifts of *E. cephalophus* were analyzed using SDMToolbox v2.4 together with ArcGIS 10.8.2. A step‐by‐step work process was developed for this study: (1) Conversion of model output: The ASCII output from MaxEnt was first converted into geospatial layers using the format converter tool in SDMToolbox; (2) Reclassification of suitability indices: The continuous suitability indices were reclassified into discrete categories using the Quick Reclassify tool in ArcGIS; (3) Geometric centroid calculation: The geometric centroids of suitable habitats were computed at multiple temporal granularities by applying geographic coordinate transformations; (4) Quantification of centroid displacement: The geometric centroid coordinates of suitable habitats were calculated across different periods via the spatial statistics tool. Centroid shifts (distance and direction) were quantified by converting geographic coordinates, thereby enabling the assessment of climate change impacts on species distribution intensity.

## 3 Results

### 3.1 Model Optimization and Accuracy Evaluation

Using 103 occurrence points and 11 environmental variables, the initial model configuration (FC = LQHPT, RM = 1, default parameters) yielded ΔAICc = 208.06. Parameter optimization via the Kuenm package identified the optimal combination (FC = QH, RM = 2.5) from 248 candidate configurations, achieving ΔAICc = 0 (Figure S1). Ten replicate runs under current climate conditions produced an AUC value of 0.977 ± 0.002 (Figure S2), demonstrating exceptional predictive performance (AUC > 0.9) for reconstructing *E. cephalophus* habitat suitability across China.

### 3.2 Key Environmental Variables Influencing *E. cephalophus* Distribution

Based on the jackknife test results (Figure S3), the primary environmental variables affecting habitat suitability include Bio12 (annual precipitation, 33.2% contribution), Ele (elevation, 18.7%), Slo (slope, 14.5%), Bio7 (temperature annual range, 9.2%), NDVI (7.9%), and Bio4 (temperature seasonality, 7.5%), collectively accounting for 91% of the total contribution rate and 73.5% cumulative permutation importance. When the suitability index exceeds 0.5, the optimal ranges for these variables are defined as follows (Table 1): Bio12 (727.79–1515.49 mm), Ele (1198.56–3612.90 m), Slo (> 25.19°), Bio7 (25.93°C–29.76°C), NDVI (0.68–0.89), and Bio4 (591.83°C–772.18°C).

### 3.3 Current and Future Potential Suitable Habitats for *E. cephalophus*

Under current climatic conditions, the primary suitable habitats for *E. cephalophus* are concentrated in the mountainous regions of Sichuan‐Guizhou‐Yunnan and the Qinba‐Wuling ranges, specifically including southern Gansu, southern Shaanxi, central Sichuan, southwestern Hubei, southeastern Chongqing, most of Guizhou, southeastern Tibet, and northwestern Yunnan (Figure 3). Additionally, fragmented habitats exist in border areas between Hunan‐Guangxi‐Guangdong and Fujian‐Zhejiang. The total suitable habitat area spans approximately 145.98 × 10^4^ km^2^ (15.21% of China's land area), with high‐, moderate‐, and low‐suitability zones covering 20.62 × 10^4^ km^2^ (2.15%), 38.85 × 10^4^ km^2^ (4.04%), and 86.51 × 10^4^ km^2^ (9.02%), respectively (Table 2).

**TABLE 2:** Projected changes in suitable habitat area of *E. cephalophus* under current conditions and future climate scenarios.

Scenario	Period	Highly suitable area (×10^4^/km^2^)	Change (%)	Moderately suitable area (×10^4^/km^2^)	Change (%)	Low suitable area (×10^4^/km^2^)	Change (%)	Total area (×10^4^/km^2^)	Total change (%)
Current	1970–2000	20.62	—	38.85	—	86.51	—	145.98	—
SSP126	2030s	14.37	−30.31	31.82	−18.10	76.41	−11.67	122.61	−16.01
	2050s	11.39	−44.76	27.27	−29.81	68.18	−21.19	106.84	−26.81
	2070s	11.53	−44.08	28.86	−25.71	73.6	−14.92	114	−21.91
	2090s	13.11	−36.42	28.60	−26.38	71.42	−17.44	113.14	−22.50
SSP245	2030s	13.88	−32.69	31.05	−20.08	74.56	−13.81	119.5	−18.14
	2050s	12.95	−37.20	29.02	−25.30	76.72	−11.32	118.68	−18.70
	2070s	10.37	−49.71	25.85	−33.46	75.55	−12.67	111.77	−23.43
	2090s	9.10	−55.87	25.04	−35.55	72.08	−16.68	106.23	−27.23
SSP370	2030s	14.24	−30.94	30.89	−20.49	75.93	−12.23	121.06	−17.07
	2050s	10.84	−47.43	26.21	−32.54	71.47	−17.39	108.52	−25.66
	2070s	6.50	−68.48	23.06	−40.64	74.13	−14.31	103.69	−28.97
	2090s	6.31	−69.40	22.02	−43.32	65.81	−23.93	94.14	−35.51
SSP585	2030s	13.40	−35.01	29.50	−24.07	82.23	−4.95	126.14	−13.59
	2050s	10.07	−51.16	25.24	−35.03	69.06	−20.17	104.37	−28.50
	2070s	6.50	−68.48	23.10	−40.54	68.33	−21.01	97.92	−32.92
	2090s	5.05	−75.51	20.15	−48.13	64.54	−25.40	89.74	−38.53

**FIGURE 5:**
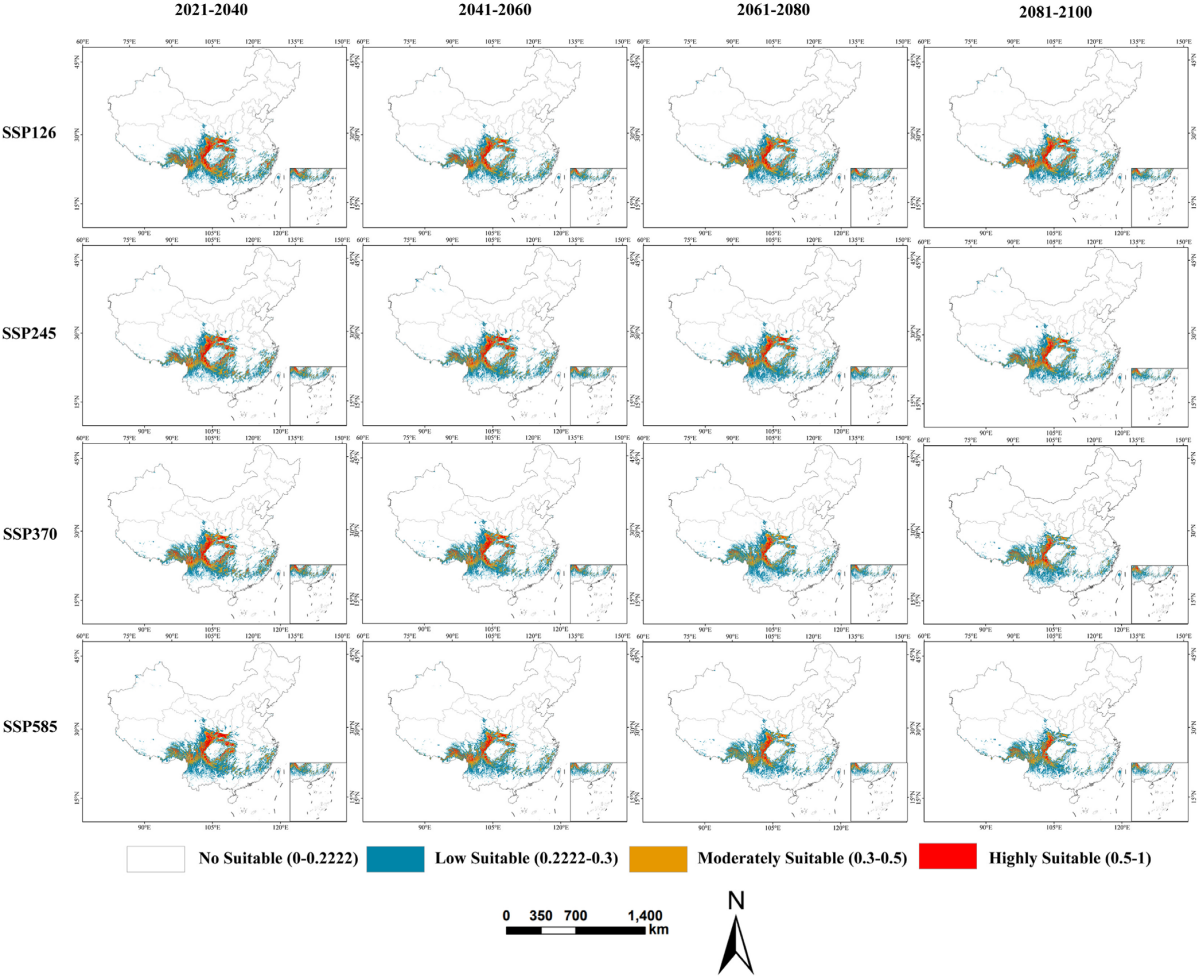
Projected distribution of *E. cephalophus* in China under future climate scenarios (2021–2100).

Future projections under four SSP scenarios (SSP126, SSP245, SSP370, SSP585) across four periods (2021–2040, 2041–2060, 2061–2080, 2081–2100) reveal a significant contraction of suitable habitats, strongly correlated with global warming intensity (Table 2; Figure 5). On average, the total suitable habitat area is projected to reduce by 21.81% (SSP126), 21.88% (SSP245), 26.80% (SSP370), and 28.39% (SSP585). This loss is most pronounced in high‐suitability areas, which decline by an average of 48.59%, while moderate‐suitability areas decrease by 31.20% and low‐suitability areas contract by 16.19%.

**FIGURE 6:**
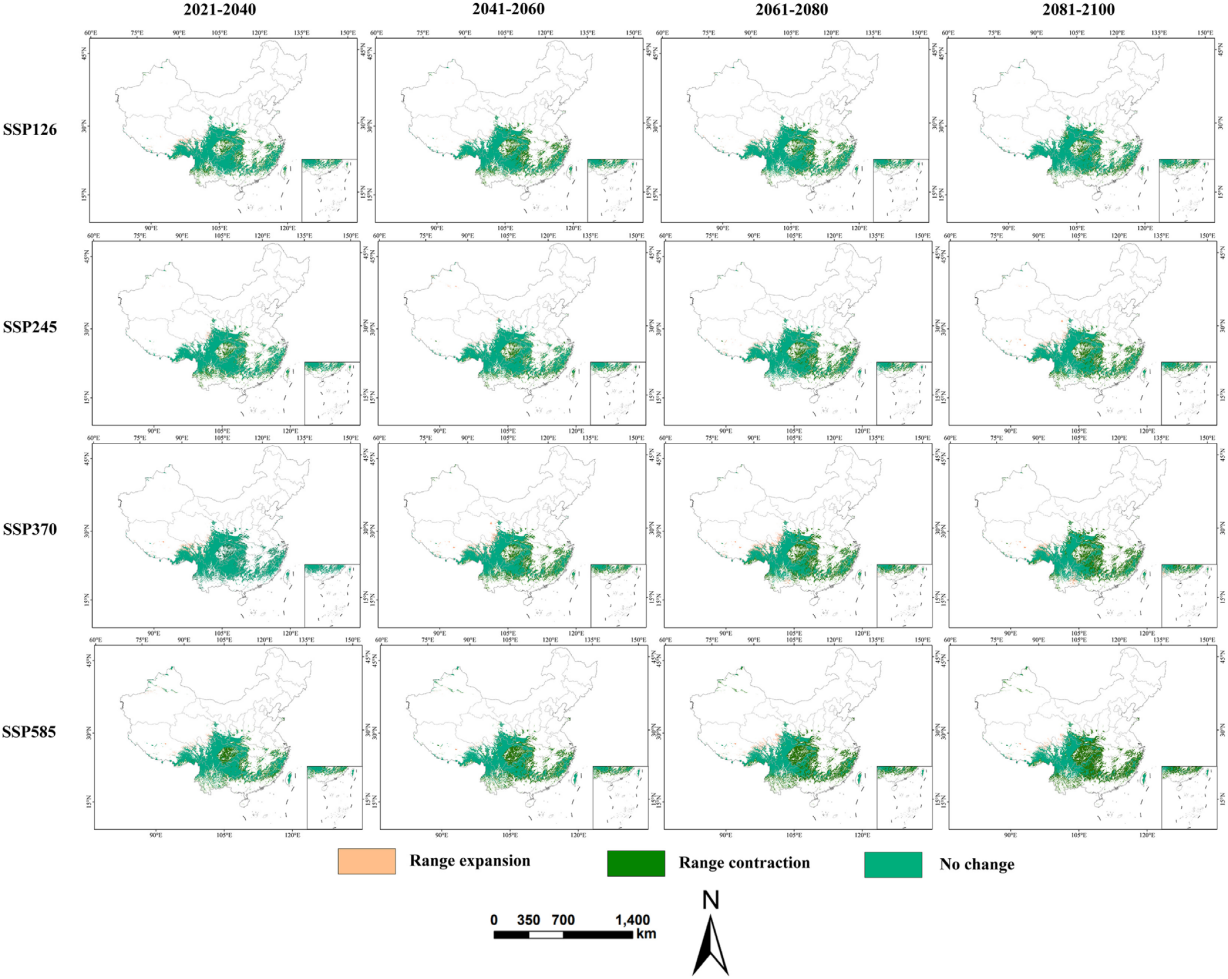
**|** Projected shifts in suitable habitats of *E. cephalophus* under current and future climate scenarios (2021–2100).

Spatially, habitat expansion is projected to occur primarily along the eastern Qinghai‐Tibet Plateau, including northwestern Sichuan, southeastern Tibet, southeastern Yunnan, and southeastern Qinghai, though these new areas are dominated by low‐ to moderate‐suitability habitats (Figure 6). In these expansion zones, the most notable gain (9.36 × 10^4^ km^2^) occurs under SSP370 during 2061–2080 (Figure 6). Conversely, significant habitat contraction is anticipated in eastern low‐elevation regions such as western Hunan, eastern Sichuan, southwestern Chongqing, central Yunnan, and central Zhejiang‐Fujian. Maximum contraction (62.67 × 10^4^ km^2^) is projected under SSP585 during 2081–2100 (Figure 6). Stable habitats, showing high persistence, are projected to remain in southwestern mountainous core areas, including most of Guizhou, southern Gansu, central Sichuan, northwestern Yunnan, and southeastern Tibet.

### 3.4 Centroid Shifts of *E. cephalophus* Suitable Habitats Under Future Climate Scenarios

**FIGURE 7:**
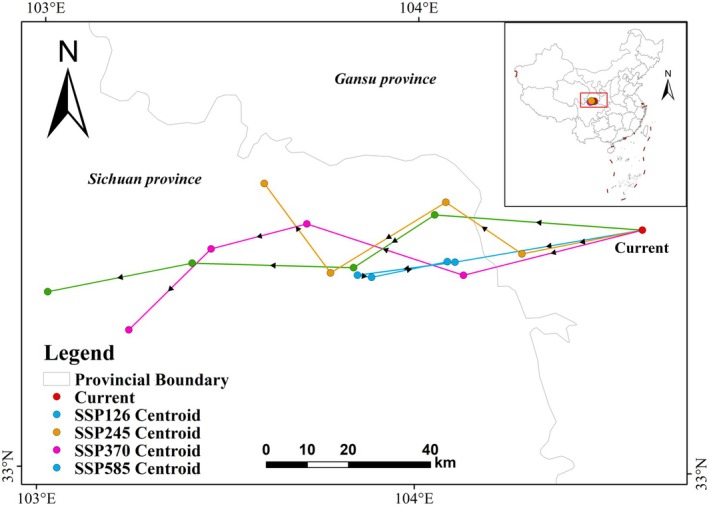
Centroid shifts of suitable habitats for *E. cephalophus* under future climate scenarios.

Analysis of centroid shifts under future climate scenarios reveals a westward displacement in the distribution of *E. cephalophus* suitable habitats, with a predominant southwestward trajectory (Figure 7). Under current climate conditions, the centroid is located in the western Wudu District of Longnan City, Gansu Province (104.78° E, 33.42° N). In future scenarios, the centroid shows an overall southwestern shift, with the magnitude of the change strongly correlated with emission intensity. The most drastic displacement occurs under the SSP585 scenario, where the centroid shifts 141.8 km to Ruoergai County, Sichuan (103.44° E, 33.27° N) by the end of the century.

## 4 Discussion

### 4.1 Key Environmental Variables Influencing the Distribution of *E. cephalophus*

Climate change is reshaping species distributions globally, driving habitat contraction, population declines, and elevated extinction risks (Moritz et al. 2008; Nicholls et al. 2022). For *E. cephalophus* , six environmental variables critically shape its habitat suitability: Bio12 (Annual Precipitation), Ele (Elevation), Slo (Slope), Bio7 (Temperature Annual Range), NDVI (Normalized Difference Vegetation Index), and Bio4 (Temperature Seasonality). Among these, Bio7 emerged as the most influential variable in model performance based on Jackknife tests, underscoring temperature as a pivotal driver of its distribution.

This suggests that *E. cephalophus* is not primarily limited by mean annual temperature itself, but rather by the magnitude of temperature fluctuations throughout the year. The species' optimal habitats are characterized by a moderate annual temperature range (Bio7: 25.93°C–29.76°C) and low temperature seasonality (Bio4: 591.83–772.18). These values indicate a preference for environments that avoid climatic extremes—that is, regions without excessively hot summers or severely cold winters. This preference is likely driven by physiological and energetic mechanisms. By inhabiting regions with lower temperature variability, the species can minimize the metabolic costs associated with thermoregulation, such as heat stress during summer and cold stress in winter (Sheng and Lu 1982). Furthermore, a more stable climate often supports more consistent and reliable forage availability throughout the year, reducing periods of resource scarcity.

This climatic preference aligns with behavioral adaptations observed in the species' evolved behavioral ecology, which includes key adaptations to mitigate thermal stress. To mitigate daytime heat stress and water loss, *E. cephalophus* exhibits crepuscular activity peaks, resting during daylight and foraging at dusk and dawn (Pu et al. 2021; Zou et al. 2021; Xie et al. 2022). Seasonal activity patterns further reflect thermal sensitivity, with higher movement abundance in spring and summer—a strategy likely tied to resource optimization during periods of favorable temperatures and food availability (Jia et al. 2014; Li et al. 2021; Liu et al. 2024). These behaviors reinforce the interpretation that the species is highly adapted to, and dependent on, a specific thermal niche defined by stability rather than by high or low mean temperatures alone.

### 4.2 Future Shifts in Suitable Habitats of *E. cephalophus* Under Climate Change Scenarios

Climate acts as a critical driver of ungulate life‐history strategies, population dynamics, and migratory behaviors (Malpeli et al. 2024). Our projections reveal a consistent decline in suitable habitats for *E. cephalophus* across future climate scenarios, with habitat loss intensifying under higher greenhouse gas emissions. This reduction correlates with the increased frequency of extreme climatic events (e.g., droughts, heatwaves) under global warming, which degrade habitat quality and disrupt resource availability. Crucially, future climate projections for the species' range indicate an increase in the variables identified as most limiting: temperature annual range (Bio7) and temperature seasonality (Bio4). This means that climate change will not only increase average temperatures but will also create more extreme climatic swings, directly threatening the stable thermal niche required by *E. cephalophus* . Studies indicate that elevated temperatures and arid conditions impair plant growth (Lipiec et al. 2013; Defalque et al. 2025), thereby reducing forage quality for herbivores. Concurrently, rising CO_2_ levels delay herbivore developmental cycles while escalating metabolic demands under heat stress, exacerbating energy deficits (Hamann et al. 2021). Similar patterns of habitat contraction under warming have been documented in other cervids, underscoring a broader vulnerability of deer species to climate‐driven range shifts (Felton et al. 2024).

Habitat loss magnitudes vary markedly across emission scenarios, highlighting the direct linkage between anthropogenic emissions and habitat viability. Future suitable habitat expansion areas for *E. cephalophus* are projected to concentrate primarily along the eastern margins of the Qinghai‐Tibet Plateau, including northwestern Sichuan, southeastern Tibet, southeastern Yunnan, and southeastern Qinghai. These regions generally exhibit warm‐humid climatic conditions and complex topography, providing ideal habitats for the species. Ecologically, their climatic niches align closely with the physiological requirements of *E. cephalophus* , while topographic complexity facilitates refuge from environmental stressors, thereby promoting population expansion. However, significant habitat contraction is anticipated in eastern low‐elevation regions such as western Hunan, eastern Sichuan, southwestern Chongqing, central Yunnan, and central Zhejiang/Fujian, likely due to intensified aridity and thermal stress under future climate scenarios, which degrade habitat quality. Notably, these contraction zones often overlap with the species' historical core distribution ranges, highlighting the existential threat posed by climate change to its original habitats. Meanwhile, ecologically stable areas—including most of Guizhou, southern Gansu, central Sichuan, northwestern Yunnan, and southeastern Tibet—demonstrate high habitat suitability persistence under climate change, attributed to their moderate climatic conditions and topographically complex terrain that provides microclimatic buffering. This stability underscores their role as climate‐resilient refugia with strong ecological buffering capacity.

Conservation strategies must adopt a spatially explicit framework: These strategies, informed by our model's projections, should be detailed and actionable: (1) In expansion zones, efforts should focus on enhancing landscape connectivity by identifying key migratory corridors using least‐cost path or circuit theory analyses. Practical measures include reforesting degraded lands and promoting wildlife‐friendly infrastructure, such as underpasses, to facilitate range shifts. (2) In contraction zones, proactive habitat restoration is essential. Interventions should be targeted at regions predicted to suffer significant degradation (e.g., western Hunan, central Zhejiang) and prioritize the restoration of native understory vegetation and bamboo groves, which constitute critical forage and cover for the species. (3) Stable habitats, which will serve as critical climate refugia, must be prioritized for enhanced protection. This involves reinforcing management of existing protected areas within these zones (e.g., in Guizhou and southern Gansu), proposing new reserves to fill protection gaps, and implementing adaptive management plans that explicitly monitor population responses to ongoing climate change.

The centroid shift trend of suitable habitats for *E. cephalophus* shows an overall westward displacement across different greenhouse gas emission scenarios, which indicates that climate change exerts more pronounced ecological impacts on western China compared to the eastern regions. This phenomenon is closely linked to China's diverse topographic landscape (Hurlbert and Jetz 2007): the prevalence of mountains and plateaus in the west drives the species to adapt to warming through vertical migration (upward shifts to higher elevations), consistent with global patterns of species range shifts toward northern latitudes or higher altitudes (Mason et al. 2014; Vacquié‐Garcia et al. 2024; Zhu et al. 2024). Mechanistically, climate warming directly alters thermal thresholds and resource distribution (e.g., bamboo and understory vegetation) in the species' habitat, forcing populations to migrate toward cooler, resource‐rich areas. Concurrently, the topographic complexity of western mountain systems provides persistent refugia through microclimatic buffering effects—such as cold‐air pooling in valleys and slope‐aspect temperature gradients—which mitigate large‐scale warming pressures and enhance population resilience to extreme climatic events (Hannah et al. 2002).

### 4.3 Strengths and Limitations of the Modeling Approach

The robustness of this study is grounded in the use of a Kuenm‐optimized MaxEnt model. This approach moves beyond default parameters, systematically calibrating the model to select the optimal configuration (RM = 2.5, FC = QH) from 248 candidates, thereby enhancing predictive accuracy and mitigating the risk of overfitting (Cobos et al. 2019; Cedano Giraldo and Mumcu Kucuker 2024). MaxEnt's proven performance in small‐sample scenarios makes it a cornerstone for predicting habitats of endangered species and projecting climate‐driven range shifts (Phillips et al. 2006; Lu et al. 2024; Yang, Ding, and Tian 2024). The utility of this Kuenm‐enhanced method is supported by numerous studies predicting range shifts for endangered species (Alanís‐Méndez et al. 2024), migratory species (Rodriguez‐Burgos et al. 2022), and invasive species (Moo‐Llanes 2021; Werenkraut et al. 2022). Furthermore, the alignment of our predicted core habitats with historical distribution records (Sheng and Lu 1982) validates the model's reliability and offers actionable insights for conservation.

However, despite these strengths, our model has several limitations. High AUC values alone do not guarantee infallibility. Limited sampling coverage may introduce spatial bias, as unsampled regions (e.g., peripheral or fragmented habitats) remain unrepresented. Furthermore, excluding critical variables such as vegetation type, water source proximity, and road density could constrain model comprehensiveness. Temporal mismatches between species occurrence data, many of which are historical, and contemporary climate datasets may also introduce biases in habitat suitability projections, particularly under rapidly changing climatic conditions (Anselmetto et al. 2025).

Another important limitation is the assumption of a static species‐environment relationship. Our model predicts future distributions based on the currently observed habitat preferences of *E. cephalophus* . However, species can exhibit functional responses in habitat selection, where their preferences and habitat use patterns change in response to environmental shifts and variations in habitat availability (Holbrook et al. 2019). Because our model is validated primarily across space rather than time, it cannot account for the possibility that habitats currently deemed unsuitable might become viable in the future through such adaptive behavioral shifts.

Finally, the exclusion of socioeconomic factors likely underestimates the cumulative impacts of anthropogenic pressures. These pressures, such as urban expansion, agricultural encroachment, and infrastructure development, are not static; they are projected to change and intensify in the future, often interacting synergistically with climate change to exacerbate habitat fragmentation and loss (Chen et al. 2025). To address these gaps, future research should prioritize multi‐disciplinary data integration. For instance, incorporating dynamic land‐use change scenarios (e.g., from the Land‐Use Harmonization project, LUH2) alongside climate projections would yield more realistic forecasts (Hurtt et al. 2020). High‐resolution land cover change maps (Wang, Peng, et al. 2024), combined with vegetation‐type data from species habitats, could refine habitat connectivity assessments. Additionally, long‐term field monitoring is particularly critical to validate model predictions and capture fine‐scale ecological feedbacks. Furthermore, combining genetic analyses to explore climate adaptation differences among *E. cephalophus* populations could provide more comprehensive scientific support for conservation and management strategies.

## 5 Conclusions

The distribution pattern of *E. cephalophus* is synergistically regulated by climate and topography. Specifically, its ecological niche is defined by climatic stability (e.g., low annual temperature range and seasonality), high annual precipitation, and topographic heterogeneity, with elevation and slope constituting core niche dimensions. The species exhibits strong dependencies on mid‐to‐high elevations (1198.56–3612.90 m), steep terrain (> 25.19°), and humid, thermally stable climates (annual precipitation: 727.79–1515.49 mm), revealing its specialized adaptation strategies to montane forest ecosystems. Under all climate scenarios, the total suitable habitat area is projected to decrease by 16.19%–48.59%, with high‐emission scenarios (SSP585) causing a reduction of 62.67 × 10^4^ km^2^ in high‐suitability zones by the 2090s, exposing eastern populations to habitat fragmentation risks. Overall, suitable habitats are expanding westward toward higher elevations, with southeastern Tibet and the western Sichuan Plateau emerging as future core refugia. The distribution centroid shifts westward by 130.7–141.8 km, confirming the critical role of these western highland areas as climate refugia. Conservation efforts should prioritize strengthening protection in southwestern mountainous core habitats, establishing cross‐provincial migration corridors in the Qinling‐Daba Mountains and Hengduan Mountains to mitigate habitat fragmentation, and deploying infrared camera networks for long‐term monitoring of population dynamics in key migration areas (e.g., Ruoergai‐Jiuzhaigou region in Aba Prefecture). For potential “novel suitable habitats” anticipated post‐2070s, proactive adjustments to nature reserve boundaries should incorporate southeastern Qinghai into protection planning.

This study demonstrates that parameter‐optimized MaxEnt models effectively predict climate responses of endangered species, though future improvements require integration of vegetation‐type data and socioeconomic factors to enhance predictive accuracy. However, future improvements require a shift toward more dynamic, process‐based models. Such models should not only account for climate change but also integrate the consequent temporal responses in vegetation and the evolving patterns of anthropogenic pressures (e.g., land‐use change) to enhance predictive accuracy. The findings provide quantitative foundations for developing climate‐adaptive conservation strategies, offering a paradigm for protecting endemic endangered species in montane ecosystems.

## Author Contributions

**Huilin Liu:** conceptualization (equal), investigation (equal), methodology (equal), software (equal), writing – original draft (equal), writing – review and editing (equal). **Qing Liu:** methodology (equal), software (equal), writing – review and editing (equal). **Xiaojuan Cui:** funding acquisition (equal), project administration (equal), resources (equal), writing – review and editing (equal). **Jianjun Peng:** funding acquisition (equal), project administration (equal), resources (equal), supervision (equal). **Sini Zhou:** data curation (equal), investigation (equal), validation (equal). **Fuli Wang:** investigation (equal), validation (equal). **Lizhen Zhong:** investigation (equal), visualization (equal). **Xia Wang:** investigation (equal), supervision (equal). **Haifeng Zheng:** investigation (equal), visualization (equal). **Chengzhong Yang:** investigation (equal), supervision (equal). **Ling Shen:** investigation (equal), supervision (equal). **Xudong Yuan:** investigation (equal), validation (equal). **Lixia Chen:** investigation (equal), visualization (equal). **Chenglun Zhang:** investigation (equal), validation (equal).

## Conflicts of Interest

The authors declare no conflicts of interest.

## Supporting information


**Appendix S1:** ece372194‐sup‐0001‐AppendixS1.csv.

**Appendix S2:** ece372194‐sup‐0002‐AppendixS2.docx.

## Data Availability

All data are in the main text and uploaded as Supporting Information, with the exception of specific location data. These location data cannot be made publicly available due to contractual obligations of the funding project and the protected status of *Elaphodus cephalophus* .

## References

[ece372194-bib-0001] Alanís‐Méndez, J. L. , V. Soto , and F. Limón‐Salvador . 2024. “Effects of Climate Change on the Distribution of Prosthechea Mariae (Orchidaceae) and Within Protected Areas in Mexico.” Plants (Basel, Switzerland) 13, no. 6: 839. 10.3390/plants13060839. 38592902 PMC10974806

[ece372194-bib-0002] Anselmetto, N. , D. Morresi , S. Barbarino , N. Loglisci , M. G. Betts , and M. Garbarino . 2025. “Species Distribution Models Built With Local Species Data Perform Better for Current Time, but Suffer From Niche Truncation.” Agricultural and Forest Meteorology 362: 110361. 10.1016/j.agrformet.2024.110361.

[ece372194-bib-0003] Burgos, J. M. , L. Buhl‐Mortensen , P. Buhl‐Mortensen , et al. 2020. “Predicting the Distribution of Indicator Taxa of Vulnerable Marine Ecosystems in the Arctic and Sub‐Arctic Waters of the Nordic Seas.” Frontiers in Marine Science 7: 131. 10.3389/fmars.2020.00131.

[ece372194-bib-0004] Cedano Giraldo, D. , and D. Mumcu Kucuker . 2024. “Ecological Niche Modeling of Lactarius Deliciosus Using Kuenm R Package: Insights Into Habitat Preferences.” Fungal Biology 128, no. 6: 2022–2031. 10.1016/j.funbio.2024.07.010. 39174237

[ece372194-bib-0005] Chen, K. , L. Ma , W. Jiang , et al. 2025. “Anthropogenic Disturbance and Climate Change Impacts on the Suitable Habitat of Sphenomorphus Incognitus in China.” Ecology and Evolution 15, no. 1: e70848. 10.1002/ece3.70848. 39839339 PMC11748457

[ece372194-bib-0006] Cobos, M. E. , A. T. Peterson , N. Barve , and L. Osorio‐Olvera . 2019. “Kuenm: An R Package for Detailed Development of Ecological Niche Models Using Maxent.” PeerJ 7: e6281. 10.7717/peerj.6281. 30755826 PMC6368831

[ece372194-bib-0007] Defalque, C. , J. Laeremans , J. Drugmand , et al. 2025. “Drought and High Temperatures Impact the Plant‐Pollinator Interactions in *Fagopyrum esculentum* .” Plants 14, no. 1: 131. 10.3390/plants14010131. 39795391 PMC11722719

[ece372194-bib-0008] Di Marco, M. , O. Venter , H. P. Possingham , and J. E. M. Watson . 2018. “Changes in Human Footprint Drive Changes in Species Extinction Risk.” Nature Communications 9, no. 1: 4621. 10.1038/s41467-018-07049-5. PMC6218474 30397204

[ece372194-bib-0009] Felton, A. M. , H. K. Wam , Z. Borowski , et al. 2024. “Climate Change and Deer in Boreal and Temperate Regions: From Physiology to Population Dynamics and Species Distributions.” Global Change Biology 30, no. 9: e17505. 10.1111/gcb.17505. 39319472

[ece372194-bib-0010] Glennon, M. J. , S. F. Langdon , M. A. Rubenstein , and M. S. Cross . 2019. “Relative Contribution of Climate and Non‐Climate Drivers in Determining Dynamic Rates of Boreal Birds at the Edge of Their Range.” PLoS One 14, no. 10: e0224308. 10.1371/journal.pone.0224308. 31648274 PMC6812788

[ece372194-bib-0011] Hamann, E. , C. Blevins , S. J. Franks , M. I. Jameel , and J. T. Anderson . 2021. “Climate Change Alters Plant‐Herbivore Interactions.” New Phytologist 229, no. 4: 1894–1910. 10.1111/nph.17036. 33111316

[ece372194-bib-0012] Hannah, L. , G. F. Midgley , and D. Millar . 2002. “Climate Change‐Integrated Conservation Strategies.” Global Ecology and Biogeography 11, no. 6: 485–495. 10.1046/j.1466-822X.2002.00306.x.

[ece372194-bib-0013] Holbrook, J. D. , L. E. Olson , N. J. DeCesare , M. Hebblewhite , J. R. Squires , and R. Steenweg . 2019. “Functional Responses in Habitat Selection: Clarifying Hypotheses and Interpretations.” Ecological Applications 29, no. 3: e01852. 10.1002/eap.1852. 30653797

[ece372194-bib-0014] Huang, R. , C. Zhang , Y. Wen , C. Wu , H. Lu , and B. Zhao . 2022. “Predicting the Habitats of *Achnatherum inebrians* in China Under Current (1970–2000) and Future Climate Conditions.” Acta Agrestia Sinica 30, no. 10: 2712–2720. 10.11733/j.issn.1007-0435.2022.10.021.

[ece372194-bib-0015] Hurlbert, A. H. , and W. Jetz . 2007. “Species Richness, Hotspots, and the Scale Dependence of Range Maps in Ecology and Conservation.” Proceedings of the National Academy of Sciences of the United States of America 104, no. 33: 13384–13389. 10.1073/pnas.0704469104. 17686977 PMC1948922

[ece372194-bib-0016] Hurtt, G. C. , L. Chini , R. Sahajpal , et al. 2020. “Harmonization of Global Land Use Change and Management for the Period 850–2100 (LUH2) for CMIP6.” Geoscientific Model Development 13, no. 11: 5425–5464. 10.5194/gmd-13-5425-2020.

[ece372194-bib-0017] Jia, X. , X. Liu , X. Yang , et al. 2014. “Seasonal Activity Patterns of Ungulates in Qinling Mountains Based on Camera‐Trap Data.” Biodiversity Science 22, no. 6: 737–745. 10.3724/SP.J.1003.2014.140073.

[ece372194-bib-0018] Khattak, R. H. , S. Ahmad , T. Mehmood , et al. 2024. “Insights Into Population Status and Habitat Patches of Conservation Concern for the Endangered Indian Pangolin ( *Manis crassicaudata* ) in Nowshera District, Northwestern Pakistan.” Biology 13, no. 9: 727. 10.3390/biology13090727. 39336154 PMC11428489

[ece372194-bib-0019] Kong, W. Y. , X. H. Li , and H. F. Zou . 2019. “Optimizing MaxEnt Model in the Prediction of Species Distribution.” Ying Yong Sheng Tai Xue Bao = the Journal of Applied Ecology 30, no. 6: 2116–2128. 10.13287/j.1001-9332.201906.029. 31257787

[ece372194-bib-0020] Lenoir, J. , J. C. Gégout , P. A. Marquet , P. de Ruffray , and H. Brisse . 2008. “A Significant Upward Shift in Plant Species Optimum Elevation During the 20th Century.” Science 320, no. 5884: 1768–1771. 10.1126/science.1156831. 18583610

[ece372194-bib-0021] Li, P. , Z. J. Zhang , H. Yang , et al. 2021. “Study on the Activity Rhythms of Ungulates in Daxiangling Nature Reserve Based on Infrared Camera Trapping.” Journal of Sichuan Forestry Science and Technology 42, no. 3: 18–23. 10.12172/202012170001.

[ece372194-bib-0022] Lipiec, J. , C. Doussan , A. Nosalewicz , and K. Kondracka . 2013. “Effect of Drought and Heat Stresses on Plant Growth and Yield: A Review.” International Agrophysics 27, no. 4: 463–477. 10.2478/intag-2013-0017.

[ece372194-bib-0023] Liu, C. , G. Newell , and M. White . 2016. “On the Selection of Thresholds for Predicting Species Occurrence With Presence‐Only Data.” Ecology and Evolution 6, no. 1: 337–348. 10.1002/ece3.1878. 26811797 PMC4716501

[ece372194-bib-0024] Liu, M. , B. Q. Zhu , Y. J. Wang , et al. 2021. “Activity Rhythm and Seasonal Changes of *Elaphodus cephalophus* in Baihe National Nature Reserve, Sichuan Province.” Journal of Sichuan Forestry Science and Technology 42, no. 2: 27–32.

[ece372194-bib-0025] Liu, Q. , T. Tao , L. Jianjun , K. Zujie , Y. Guiqing , and Y. Cuncun . 2024. “Assessment of the Suitable Habitat for the Tufted Deer (*Elaphodus cephalophus*) in the Hupingshan National Nature Reserve.” China Environmental Science 44, no. 5: 2619–2629. 10.19674/j.cnki.issn1000-6923.20240025.001.

[ece372194-bib-0026] Lu, J. , X. Zhang , and J. Wang . 2023. “Daily Activity Rhythms of Muntiacus Reevesi and *Elaphodus cephalophus* Revealed by Infrared‐Triggered Cameras.” Shaanxi Forest Science and Technology 51, no. 1: 89–92.

[ece372194-bib-0027] Lu, K. , M. Liu , Q. Feng , W. Liu , M. Zhu , and Y. Duan . 2024. “Predicting the Global Distribution of Nitraria L. Under Climate Change Based on Optimized MaxEnt Modeling.” Plants 14, no. 1: 67. 10.3390/plants14010067. 39795327 PMC11722589

[ece372194-bib-0028] MacPherson, J. , P. Wright , N. Schumaker , et al. 2024. “Use of Multi‐Modelling Methods to Inform Conservation and Reintroductions of Pine Marten *Martes martes* in Britain.” Stacks (Portland, Or.) 2024: 24004. 10.60102/stacks-24004. 39744334 PMC11684397

[ece372194-bib-0029] Malpeli, K. C. , S. C. Endyke , S. R. Weiskopf , et al. 2024. “Existing Evidence on the Effects of Climate Variability and Climate Change on Ungulates in North America: A Systematic Map.” Environmental Evidence 13: 8. 10.1186/s13750-024-00331-8. 39294746 PMC11378825

[ece372194-bib-0030] Mason, T. H. E. , P. A. Stephens , M. Apollonio , and S. G. Willis . 2014. “Predicting Potential Responses to Future Climate in an Alpine Ungulate: Interspecific Interactions Exceed Climate Effects.” Global Change Biology 20, no. 12: 3872–3882. 10.1111/gcb.12641. 24957266

[ece372194-bib-0031] Moo‐Llanes, D. A. 2021. “Inferring Distributional Shifts of Asian Giant Hornet Vespa Mandarinia Smith in Climate Change Scenarios.” Neotropical Entomology 50, no. 4: 673–676. 10.1007/s13744-020-00840-4. 33555561

[ece372194-bib-0032] Morales, N. S. , I. C. Fernández , and V. Baca‐González . 2017. “Maxent's Parameter Configuration and Small Samples: Are We Paying Attention to Recommendations? A Systematic Review.” PeerJ 5: e3093. 10.7717/peerj.3093. 28316894 PMC5354112

[ece372194-bib-0033] Moritz, C. , J. L. Patton , C. J. Conroy , J. L. Parra , G. C. White , and S. R. Beissinger . 2008. “Impact of a Century of Climate Change on Small‐Mammal Communities in Yosemite National Park, USA.” Science (New York, N.Y.) 322, no. 5899: 261–264. 10.1126/science.1163428. 18845755

[ece372194-bib-0034] Neves, J. M. M. , V. S. Belo , C. M. S. Catita , B. F. A. Oliveira , and M. A. P. Horta . 2024. “Modeling of Human Rabies Cases in Brazil in Different Future Global Warming Scenarios.” International Journal of Environmental Research and Public Health 21, no. 2: 212. 10.3390/ijerph21020212. 38397701 PMC10888213

[ece372194-bib-0035] Nicholls, Z. , M. Meinshausen , J. Lewis , et al. 2022. “Changes in IPCC Scenario Assessment Emulators Between SR1.5 and AR6 Unraveled.” Geophysical Research Letters 49, no. 20: e2022GL099788. 10.1029/2022GL099788. PMC9788315 36589268

[ece372194-bib-0036] Peng, S. , Q. Li , and H. Ren . 2002. “Impact of Climate Change on Wildlife.” Acta Ecologica Sinica 7: 1153–1159.

[ece372194-bib-0037] Phillips, S. J. , R. P. Anderson , and R. E. Schapire . 2006. “Maximum Entropy Modeling of Species Geographic Distributions.” Ecological Modelling 190, no. 3: 231–259. 10.1016/j.ecolmodel.2005.03.026.

[ece372194-bib-0038] Poudel, A. , P. Adhikari , S. H. Choi , J. Y. Yun , Y. H. Lee , and S. H. Hong . 2024. “Predicting the Invasion Risk of the Highly Invasive *Acacia mearnsii* in Asia Under Global Climate Change.” Plants Basel, Switzerland 13, no. 20: 2846. 10.3390/plants13202846. 39458793 PMC11510992

[ece372194-bib-0039] Pu, D. , Y. Chen , R. Zhang , et al. 2021. “Activity Rhythms of Tufted Deer (*Elaphodus cephalophus*) in the Meigu Dafengding National Nature Reserve, Sichuan, China.” Journal of Pu'er University 37, no. 3: 1–5.

[ece372194-bib-0040] Radosavljevic, A. , and R. P. Anderson . 2014. “Making Better Maxent Models of Species Distributions: Complexity, Overfitting and Evaluation.” Journal of Biogeography 41, no. 4: 629–643. 10.1111/jbi.12227.

[ece372194-bib-0041] Rodriguez‐Burgos, A. M. , F. J. Briceño‐Zuluaga , J. L. Ávila Jiménez , et al. 2022. “The Impact of Climate Change on the Distribution of *Sphyrna lewini* in the Tropical Eastern Pacific.” Marine Environmental Research 180: 105696. 10.1016/j.marenvres.2022.105696. 35932509

[ece372194-bib-0042] Root, T. L. , J. T. Price , K. R. Hall , S. H. Schneider , C. Rosenzweig , and J. A. Pounds . 2003. “Fingerprints of Global Warming on Wild Animals and Plants.” Nature 421, no. 6918: 57–60. 10.1038/nature01333. 12511952

[ece372194-bib-0043] Sheng, H. , and H. Lu . 1982. “Distribution, Habits and Resource Status of the Tufted Deer (*Elaphodus cephalophus*).” Current Zoology 28, no. 3: 307–311.

[ece372194-bib-0044] Sheng, H. , and T. Wu . 1981. “Resources of Black Muntjac, Reeve's Muntjac, Tufted Deer, and Sika Deer in the Western Mountains of Zhejiang Province.” Chinese Journal of Wildlife 2: 33–34. 10.19711/j.cnki.issn2310-1490.1981.02.015.

[ece372194-bib-0045] Shi, X. , J. Wang , L. Zhang , et al. 2023. “Prediction of the Potentially Suitable Areas of *Litsea cubeba* in China Based on Future Climate Change Using the Optimized MaxEnt Model.” Ecological Indicators 148: 110093. 10.1016/j.ecolind.2023.110093. PMC10585429 37869439

[ece372194-bib-0046] Shirzad, R. , A. A. Alesheikh , M. Asgharzadeh , B. Hoseini , and A. Lotfata . 2023. “Spatio‐Temporal Modeling of Human Leptospirosis Prevalence Using the Maximum Entropy Model.” BMC Public Health 23, no. 1: 2521. 10.1186/s12889-023-17391-z. 38104062 PMC10724969

[ece372194-bib-0047] Swets, J. A. 1988. “Measuring the Accuracy of Diagnostic Systems.” Science 240, no. 4857: 1285–1293. 10.1126/science.3287615. 3287615

[ece372194-bib-0048] Syfert, M. M. , M. J. Smith , and D. A. Coomes . 2013. “The Effects of Sampling Bias and Model Complexity on the Predictive Performance of MaxEnt Species Distribution Models.” PLoS One 8, no. 2: e55158. 10.1371/journal.pone.0055158. 23457462 PMC3573023

[ece372194-bib-0049] Vacquié‐Garcia, J. , J. Spitz , M. Hammill , et al. 2024. “Foraging Habits of Northwest Atlantic Hooded Seals Over the Past 30 Years: Future Habitat Suitability Under Global Warming.” Global Change Biology 30, no. 3: e17186. 10.1111/gcb.17186. 38450925

[ece372194-bib-0050] Waheed, M. , S. M. Haq , F. Arshad , et al. 2024. “ *Xanthium strumarium* L., an Invasive Species in the Subtropics: Prediction of Potential Distribution Areas and Climate Adaptability in Pakistan.” BMC Ecology and Evolution 24, no. 1: 124. 10.1186/s12862-024-02310-6. 39390368 PMC11465908

[ece372194-bib-0051] Walther, G.‐R. , E. Post , P. Convey , et al. 2002. “Ecological Responses to Recent Climate Change.” Nature 416, no. 6879: 389–395. 10.1038/416389a. 11919621

[ece372194-bib-0052] Wang, D. , Q. Peng , X. Li , et al. 2024. “A Long‐Term High‐Resolution Dataset of Grasslands Grazing Intensity in China.” Scientific Data 11, no. 1: 1194. 10.1038/s41597-024-04045-x. 39500911 PMC11538541

[ece372194-bib-0053] Wang, P. , G. Liu , X. Li , et al. 2024. “Potential Distribution of *Elymus nutans* in China Under Future Climate Scenarios.” Chinese Journal of Ecology 44: 1–15.

[ece372194-bib-0054] Werenkraut, V. , M. P. Arbetman , and P. N. Fergnani . 2022. “The Oriental Hornet (*Vespa orientalis* L.): A Threat to the Americas?” Neotropical Entomology 51, no. 2: 330–338. 10.1007/s13744-021-00929-4. 34873676

[ece372194-bib-0055] Wu, T. , Y. Lu , Y. Fang , et al. 2019. “The Beijing Climate Center Climate System Model (BCC‐CSM): The Main Progress From CMIP5 to CMIP6.” Geoscientific Model Development 12, no. 4: 1573–1600. 10.5194/gmd-12-1573-2019.

[ece372194-bib-0056] Xie, B. , C. Wang , H. Fan , and B. Meng . 2022. “Activity Rhythm Analysis of Sympatric Elaphodus Cephalophus and *Muntiacus reevesi* Based on Camera‐Traps in the Fanjingshan Mountains.” Sichuan Journal of Zoology 41, no. 2: 175–183. 10.11984/j.issn.1000-7083.20210210.

[ece372194-bib-0057] Yang, J. , G. Ding , and X. Tian . 2024. “Research Progress on the Application of the MaxEnt Model in Species Habitat Prediction.” Chinese Journal of Applied Ecology 36: 1–12. 10.13287/j.1001-9332.202502.025. 40370179

[ece372194-bib-0058] Yang, M. , M. Yang , Z. Deng , et al. 2024. “Habitat Suitability Assessment of *Elaphodus cephalophus* in Hunan Province Based on MaxEnt Model.” Chinese Journal of Ecology 44: 1–10.

[ece372194-bib-0059] Yang, Z. , Y. Wei , L. Guo , et al. 2025. “Predicting the Distribution of Potential Habitat Areas of *Ageratina adenophora* in China Based on an Optimised Maxent Model.” Journal of Inner Mongolia Agricultural University(Natural Science Edition) 46, no. 1: 49–56. 10.16853/j.cnki.1009-3575.2025.01.007.

[ece372194-bib-0060] Yao, W. , Z. Wang , Y. Fan , et al. 2025. “Prediction of Potential Habitat Distributions and Climate Change Impacts on the Rare Species Woonyoungia Septentrionalis (Magnoliaceae) in China Based on MaxEnt.” Plants 14, no. 1: 86. 10.3390/plants14010086. PMC11723205 39795346

[ece372194-bib-0061] Zhang, C. , Y. Chen , B. Xu , Y. Xue , and Y. Ren . 2020. “Improving Prediction of Rare Species’ Distribution From Community Data.” Scientific Reports 10: 12230. 10.1038/s41598-020-69157-x. 32699354 PMC7376031

[ece372194-bib-0062] Zhang, Z. , and F. Wei . 2007. “Winter Habitat Selection by Tufted Deer in Fengtongzhai Nature Reserve.” Journal of China West Normal University (Natural Sciences) 1: 1–6, 10. 10.16246/j.issn.1673-5072.2007.01.001.

[ece372194-bib-0063] Zhu, Y. , X. Cui , B. Kang , et al. 2024. “Comparative Analysis of Climate‐Induced Changes in Distribution of Representative Fish Species in the Yellow Sea.” Science of the Total Environment 912: 168699. 10.1016/j.scitotenv.2023.168699. 38008324

[ece372194-bib-0064] Zhuang, X. , C. Wang , W. Hong , et al. 2024. “Potential Suitable Habitats of *Chelydra serpentina* Linnaeus in China Under Future Climate Projection, Based on MaxEnt and ArcGIS.” Journal of Biosafety 33, no. 4: 393–401, 422. 10.3969/j.issn.2095-1787.2024.04.010.

[ece372194-bib-0065] Zou, Q. , C. Peng , X. Yang , and G. Li . 2021. “Spatiotemporal Pattern of Co‐Existence of Two Sympatric Cervid Species in Mayanghe National Nature Reserve, Guizhou, China.” Chinese Journal of Wildlife 42, no. 1: 5–13. 10.19711/j.cnki.issn2310-1490.2021.01.001.

