# Supplementary material for: Prediction of Potential Suitable Habitats for Elaphodus cephalophus in China Under Climate Change Scenarios
Source: Ecol Evol. 2025 Sep 25;15(10):e72194. doi: 10.1002/ece3.72194 (PMC12463574; doi:10.1002/ece3.72194)
Supplement: Supplementary file 2 — Appendix S2: ece372194‐sup‐0002‐AppendixS2.docx. [file ECE3-15-e72194-s002.docx]

Supplementary Materials

1. Supplementary Figures

| 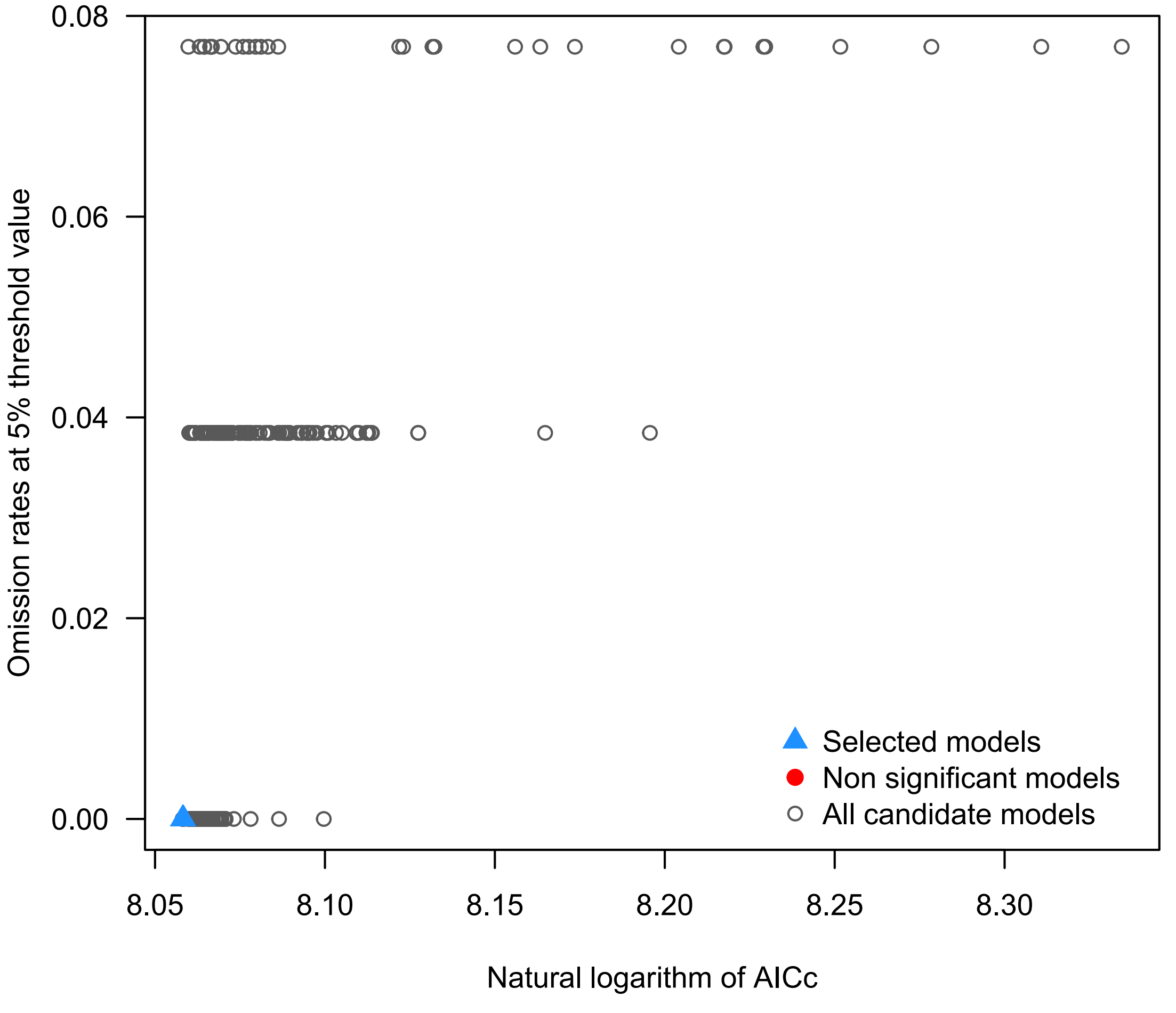 |
| --- |

**Figure S1 |** Distribution of all models, non-statistically significant models, and selected models in terms of AICc and omission rate values.

| 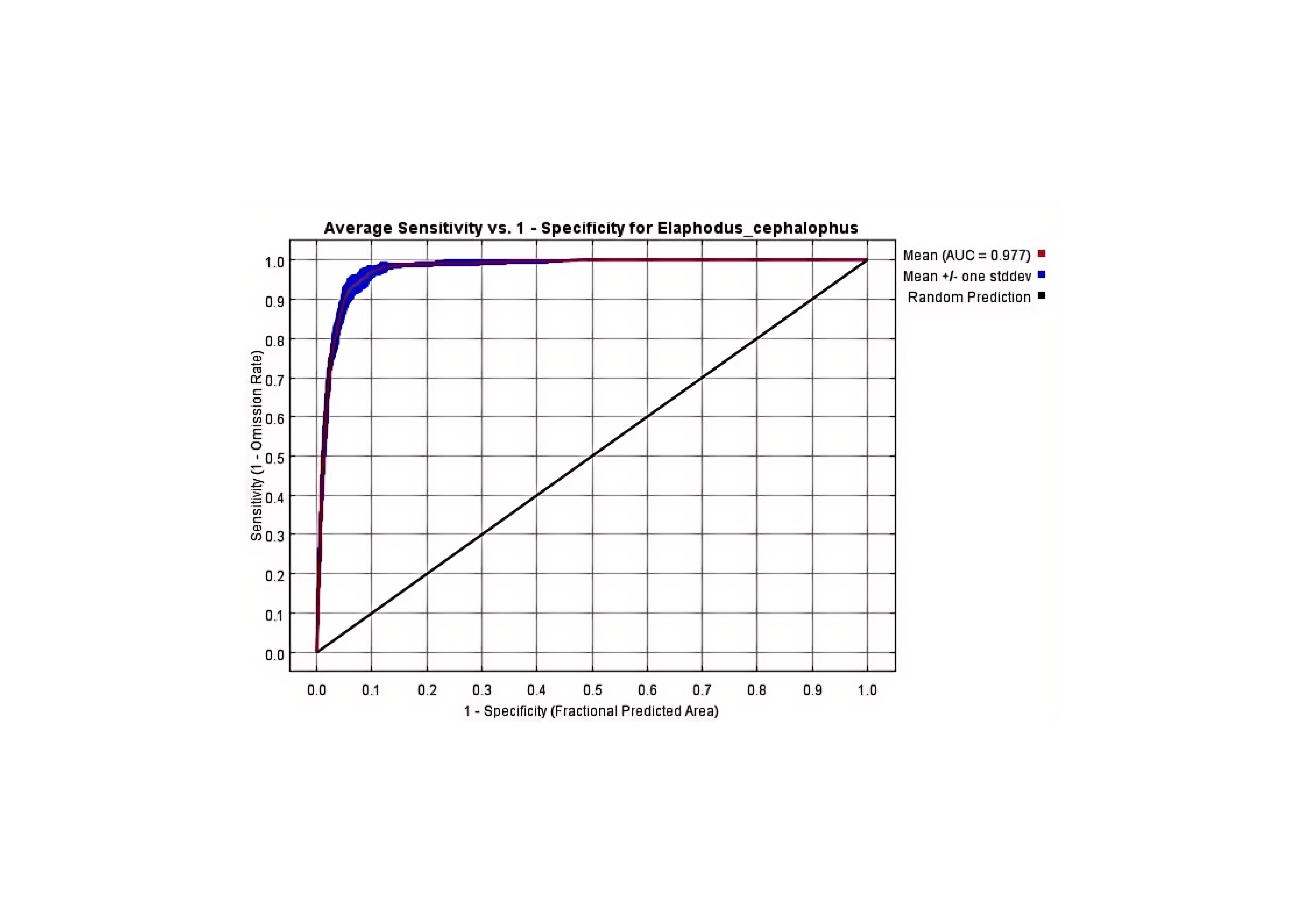 |
| --- |

**Figure S2 |** The receiver operating characteristic (ROC) curve for the same data, again averaged over the replicate runs. The average training AUC for the replicate runs is 0.977, and the standard deviation is 0.002.

| 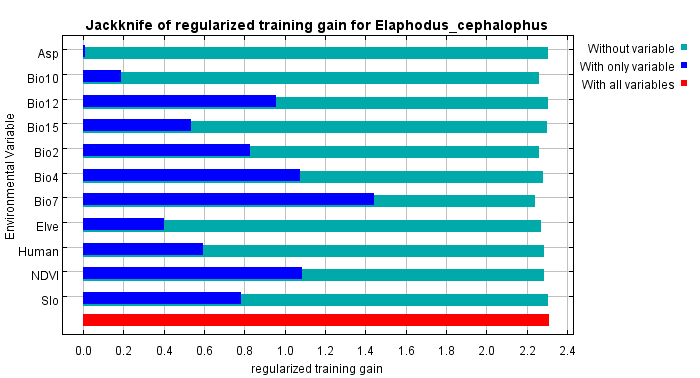 |
| --- |

**Figure S3 |** The results of the jackknife test of variable importance. The environmental variable with highest gain when used in isolation is Bio7, which therefore appears to have the most useful information by itself. The environmental variable that decreases the gain the most when it is omitted is Bio7, which therefore appears to have the most information that isn't present in the other variables. Values shown are averages over replicate runs.

1. Supplementary Tables

**Table S1** |**Pearson Correlation Matrix of 19 Environmental Variables for MaxEnt Modeling**

|  | Bio1 | Bio2 | Bio3 | Bio4 | Bio5 | Bio6 | Bio7 | Bio8 | Bio9 | Bio10 | Bio11 | Bio12 | Bio13 | Bio14 | Bio15 | Bio16 | Bio17 | Bio18 | Bio19 |
| --- | --- | --- | --- | --- | --- | --- | --- | --- | --- | --- | --- | --- | --- | --- | --- | --- | --- | --- | --- |
| Bio1 | 1 | -0.655 | -0.584 | 0.448 | 0.969 | 0.984 | -0.303 | 0.954 | 0.965 | 0.98 | 0.979 | 0.633 | 0.656 | 0.562 | -0.317 | 0.664 | 0.546 | 0.634 | 0.509 |
| Bio2 | -0.655 | 1 | 0.945 | -0.662 | -0.673 | -0.738 | 0.366 | -0.668 | -0.536 | -0.726 | -0.559 | -0.684 | -0.516 | -0.612 | 0.571 | -0.58 | -0.562 | -0.575 | -0.485 |
| Bio3 | -0.584 | 0.945 | 1 | -0.855 | -0.679 | -0.633 | 0.051 | -0.638 | -0.413 | -0.706 | -0.439 | -0.609 | -0.399 | -0.57 | 0.554 | -0.462 | -0.52 | -0.449 | -0.432 |
| Bio4 | 0.448 | -0.662 | -0.855 | 1 | 0.629 | 0.418 | 0.437 | 0.568 | 0.238 | 0.618 | 0.26 | 0.398 | 0.18 | 0.415 | -0.462 | 0.219 | 0.39 | 0.209 | 0.301 |
| Bio5 | 0.969 | -0.673 | -0.679 | 0.629 | 1 | 0.938 | -0.089 | 0.963 | 0.884 | 0.993 | 0.905 | 0.592 | 0.562 | 0.538 | -0.353 | 0.575 | 0.521 | 0.546 | 0.47 |
| Bio6 | 0.984 | -0.738 | -0.633 | 0.418 | 0.938 | 1 | -0.43 | 0.934 | 0.953 | 0.96 | 0.97 | 0.656 | 0.659 | 0.576 | -0.364 | 0.678 | 0.553 | 0.653 | 0.514 |
| Bio7 | -0.303 | 0.366 | 0.051 | 0.437 | -0.089 | -0.43 | 1 | -0.174 | -0.433 | -0.172 | -0.427 | -0.342 | -0.431 | -0.252 | 0.126 | -0.45 | -0.229 | -0.453 | -0.252 |
| Bio8 | 0.954 | -0.668 | -0.638 | 0.568 | 0.963 | 0.934 | -0.174 | 1 | 0.862 | 0.968 | 0.902 | 0.517 | 0.526 | 0.42 | -0.261 | 0.537 | 0.393 | 0.561 | 0.333 |
| Bio9 | 0.965 | -0.536 | -0.413 | 0.238 | 0.884 | 0.953 | -0.433 | 0.862 | 1 | 0.901 | 0.991 | 0.621 | 0.688 | 0.579 | -0.292 | 0.68 | 0.577 | 0.622 | 0.569 |
| Bio10 | 0.98 | -0.726 | -0.706 | 0.618 | 0.993 | 0.96 | -0.172 | 0.968 | 0.901 | 1 | 0.92 | 0.642 | 0.613 | 0.581 | -0.383 | 0.629 | 0.562 | 0.602 | 0.509 |
| Bio11 | 0.979 | -0.559 | -0.439 | 0.26 | 0.905 | 0.97 | -0.427 | 0.902 | 0.991 | 0.92 | 1 | 0.6 | 0.669 | 0.521 | -0.251 | 0.669 | 0.51 | 0.639 | 0.49 |
| Bio12 | 0.633 | -0.684 | -0.609 | 0.398 | 0.592 | 0.656 | -0.342 | 0.517 | 0.621 | 0.642 | 0.6 | 1 | 0.876 | 0.925 | -0.661 | 0.928 | 0.903 | 0.876 | 0.859 |
| Bio13 | 0.656 | -0.516 | -0.399 | 0.18 | 0.562 | 0.659 | -0.431 | 0.526 | 0.688 | 0.613 | 0.669 | 0.876 | 1 | 0.74 | -0.285 | 0.982 | 0.725 | 0.925 | 0.711 |
| Bio14 | 0.562 | -0.612 | -0.57 | 0.415 | 0.538 | 0.576 | -0.252 | 0.42 | 0.579 | 0.581 | 0.521 | 0.925 | 0.74 | 1 | -0.756 | 0.788 | 0.992 | 0.677 | 0.97 |
| Bio15 | -0.317 | 0.571 | 0.554 | -0.462 | -0.353 | -0.364 | 0.126 | -0.261 | -0.292 | -0.383 | -0.251 | -0.661 | -0.285 | -0.756 | 1 | -0.367 | -0.766 | -0.32 | -0.725 |
| Bio16 | 0.664 | -0.58 | -0.462 | 0.219 | 0.575 | 0.678 | -0.45 | 0.537 | 0.68 | 0.629 | 0.669 | 0.928 | 0.982 | 0.788 | -0.367 | 1 | 0.765 | 0.948 | 0.741 |
| Bio17 | 0.546 | -0.562 | -0.52 | 0.39 | 0.521 | 0.553 | -0.229 | 0.393 | 0.577 | 0.562 | 0.51 | 0.903 | 0.725 | 0.992 | -0.766 | 0.765 | 1 | 0.644 | 0.984 |
| Bio18 | 0.634 | -0.575 | -0.449 | 0.209 | 0.546 | 0.653 | -0.453 | 0.561 | 0.622 | 0.602 | 0.639 | 0.876 | 0.925 | 0.677 | -0.32 | 0.948 | 0.644 | 1 | 0.584 |
| Bio19 | 0.509 | -0.485 | -0.432 | 0.301 | 0.47 | 0.514 | -0.252 | 0.333 | 0.569 | 0.509 | 0.49 | 0.859 | 0.711 | 0.97 | -0.725 | 0.741 | 0.984 | 0.584 | 1 |
